# Clients’ perceptions of nursing care services at selected public health facilities in Namibia: A qualitative study

**DOI:** 10.4102/hsag.v31i0.3252

**Published:** 2026-06-11

**Authors:** Nestor Tomas, Gisela H. van Rensburg, Mokgadi Matlakala

**Affiliations:** 1 Department of Health Studies, College of Human Sciences, University of South Africa, Pretoria, South Africa; 2 School of Nursing and Public Health, Faculty of Health Sciences and Veterinary Medicine, University of Namibia, Rundu, Namibia

**Keywords:** client satisfaction, Namibia, nursing care services, public health facilities, quality of healthcare

## Abstract

**Background:**

Satisfaction is a subjective phenomenon that is influenced by various contextual factors. In the healthcare sector, establishing a therapeutic relationship with patients is essential for facilitating active, mutual engagement and fostering collaboration. However, patient satisfaction with nursing care services in Namibia remains under-researched.

**Aim:**

The study explored and described clients’ perceptions of nursing care services at selected public health facilities in Namibia.

**Setting:**

The study was conducted at two public health facilities in Namibia.

**Methods:**

An exploratory, descriptive qualitative design was utilised to recruit 13 clients for individual interviews through purposive sampling. Data were collected in 2022 using an interview guide and analysed using Braun and Clarke’s six-step thematic analysis.

**Results:**

Two key themes emerged: (1) nurse–client communication dynamics, encompassing clients’ views on client satisfaction, and (2) innovative client suggestions for enhancing client satisfaction, detailing measures to address identified challenges.

**Conclusion:**

The study’s findings underscore the need to implement targeted training initiatives for nurses and to adopt client-centred care models. Future research should explore client satisfaction through larger cross-sectional or longitudinal studies to inform strategies to enhance client satisfaction with nursing care.

**Contribution:**

The study provides valuable insights into client satisfaction and has implications for nursing practice in Namibia.

## Introduction

### Background

Nursing care services are integral to the delivery of high-quality healthcare, with a demonstrable impact on patient satisfaction (Mulugeta et al. 2019:1; Yan et al. 2022:1). As a key metric for evaluating overall patient experience, satisfaction is a crucial determinant of health outcomes and a vital component of hospital quality improvement measures (Ferreira et al. 2023:1).

The World Health Organization (WHO) has globally prioritised meeting clients’ health needs, and this objective serves as a valuable indicator of nursing performance in aligning care with patient values and expectations (Oxelmark et al. 2018:612; Wudu 2021:177). Within the nursing profession, it is essential to cultivate a therapeutic relationship with patients to facilitate active, mutual engagement and information exchange, while simultaneously fostering a collaborative, non-hierarchical dynamic. Despite its function as a tangible measure of the quality of nursing services (Chatterjee & Suy 2019:2; Yan et al. 2022:1), patient satisfaction with nursing care remains an understudied area of research in Namibia.

The WHO clinical practice guidelines require healthcare providers, including nurses, to adhere to professional and ethical standards (Lindwall & Lohne 2021:1039). Client satisfaction hinges on the ability to prioritise clients’ best interests and meet their expectations (Asamrew, Endris & Tadesse 2020:2). Clients are fundamental to the existence of all healthcare organisations; thus, the quality-of-service provision directly influences their satisfaction. However, due to its subjective nature, measuring client satisfaction is complex and influenced by various factors, including individual expectations, experiences with healthcare services, health status, quality of life, and disease (Asamrew et al. 2020:2; Okeny et al. 2024:2; Sitio & Ali 2019:552; Wudu 2021:177; Yan et al. 2022:1).

Key factors contributing to client satisfaction include perceived requirements, expectations, and overall experiences with healthcare providers (Fridell et al. 2020:2). Additionally, several elements, such as the perceived responsiveness of nurses, experiences of compassionate care, individual patient circumstances, health status, socio-demographic attributes, and context-related factors, significantly affect how clients experience satisfaction (Abidova, Da Silva & Moreira 2020:392; Mulugeta et al. 2019:2; Wudu 2021:178).

Prior research indicates that client satisfaction can be achieved through effective bedside manners, clear communication, active engagement of caregivers with patients, and the provision of clear instructions (Kannan, Avudaiappan & Annamalai 2020:471; Kaur et al. 2020:1). Clients’ perceptions of healthcare quality are significant for various reasons, including client contentment, willingness to return for further services, and adherence to medical advice (Sitio & Ali 2019:551). Failure to attain adequate levels of client satisfaction can adversely affect health quality, leading clients to deviate from their treatment plans.

Therefore, considering clients’ perspectives is crucial for fostering partnerships and reaching mutual agreements between patients and healthcare providers, all grounded in evidence-based practices (Rosengren, Brannefors & Carlstrom 2021:266). Similarly, clients’ feedback and perceptions are essential prerequisites for several accreditation and monitoring programmes applied to hospital services (Abbasi-Moghaddam et al. 2019:1; Kaur et al. 2020:1; Suhail & Srinivasulu 2021:93). Thus, satisfaction serves as a vital metric for evaluating the effectiveness of healthcare service delivery and treatment outcomes (Ninson & Morgan 2021; Tran et al. 2019).

Evidence suggests that clients’ perspectives and their active involvement in decision-making are crucial to the quality of services provided, and should therefore be regarded as a fundamental principle (Oldland et al. 2020:151). However, various studies have indicated that weaknesses, underfunding, dysfunction, and inequity in many African health systems, including those of major economies such as South Africa, Nigeria, and Kenya (Accrombessi & Cook 2023:181; Lambiris et al. 2023:1), are often linked to the exclusion of clients from decision-making processes and the overall poor quality of nursing care services. This inefficiency is also evident in many Southern African Development Community member nations, where, for example, communities are excluded from decision-making and health budget allocations fall short of the 15% Abuja Declaration mandate (Bwalya 2021:3). While, client satisfaction is shaped by various factors namely individual patient health status, circumstances, socio-demographic characteristics (Abidova et al. 2020:392; Mulugeta et al. 2019:2; Wudu 2021:178), these factors arguably may vary based on context. However, despite the importance of this area of nursing practice, research (Abidova et al. 2020:391; Adu Gyamf et al. 2023:373; Mulugeta et al. 2019:1; Yan et al. 2022:1) has predominantly focused on settings outside of Namibia, leaving an empirical gap. This study explored and described the perceptions of clients regarding nursing care services within select public health facilities in Namibia.

## Research methods and design

### Design and setting

An explorative descriptive qualitative design was employed. This design was preferred for its low-inference descriptions (Yang et al. 2024), making it well-suited for examining clients’ perceptions regarding their satisfaction with nursing care services at selected public health facilities in Namibia. The study followed the Consolidated Criteria for Reporting Qualitative Research Checklist to guarantee comprehensive documentation of the methods used (Buus & Perron 2020:1; Tong et al. 2007:1).

The study was conducted at two public health facilities in Namibia – a clinic and an intermediate state hospital located at the heart of Rundu town in the Kavango-East region, which has an estimated catchment population of 39 350 (The Government of the Republic of Namibia 2019:1). The intermediate hospital serves as a referral hospital for clients from district hospitals and clinics in Kavango-East, Kavango-West, the Zambezi region, and Angola. The public healthcare facilities’ location on the Trans-Caprivi Corridor necessitates international-standard care for its local and transit patients to ensure safety and satisfaction. Elevating these standards is also a strategic move to mitigate the surge in medical lawsuits currently burdening the Ministry of Health and Social Services (MoHSS) with millions of Namibian Dollars in legal costs and settlement claims (Kruger 2024:1; New Era Newspaper 2024:1).

### Participants recruitment and sampling

The study collected data from 13 purposively selected participants at the selected public health facilities in Namibia. As recommended by Hirose and Creswell (2023:274), this approach targeted individuals with relevant expertise regarding nursing service provision at the selected facilities. The study employed the following eligibility criteria: (1) participants who had received nursing care at the selected health facilities for a period of a year or longer; (2) mentally fit participants aged 18 years or older; and (3) willingness to participate. Participants were recruited and informed of the study’s objectives and significance upon exiting the selected health facilities. Written informed consent and permission for audio recording were obtained on the date of recruitment. During the recruitment phase, the researcher strictly adhered to Coronavirus Disease of 2019 (COVID-19) safety protocols, including the mandatory use of face masks, hand sanitisation, and the maintenance of physical distancing. Participants who were critically ill, deemed mentally unfit, or nurses on leave for a period exceeding 2 months were excluded from participation.

### Data collection

Data were collected between November 2021 and February 2022 after the first author N.T. explained the purpose, objectives, significance, and duration of the in-depth individual interviews. The use of telephone interviews was essential in light of the legal constraints and safety protocols implemented during the COVID-19 pandemic (Adom, Osei & Adu-Agyem 2020:1; Kennedy et al. 2021:1; Self 2021:1). On the scheduled date of the telephonic interview, verbal consent was obtained and recorded to reconfirm the voluntary participation of all individuals who had previously provided written informed consent. All participants were made aware of their right to withdraw from the study without facing any consequences.

Rapport was established with all study participants through engaging in ice-breaking conversations. A phone-based audio recorder was used during the data collection phase to ensure the trustworthiness of the data collected. This method gained popularity in addressing the criticism towards misquoting participants, as the recorded data can be retrieved (Rutakumwa et al. 2020:2). The central questions were: (1) *What are your views with regard to nursing care services at this health facility?* (2) *what are your suggestions to promote client satisfaction with nursing care services at this facility?* Probing questions, such as ‘What do you mean by that?’, ‘Explain more’, and ‘Could you please elaborate on your suggestion?’ were asked in English to elicit a more in-depth understanding of the phenomenon being investigated.

Given that the participants’ body language could not be captured due to COVID-19, the field notes of the telephonic interviews were exclusively based on verbal cues, such as the tone of language, the quickness of the responses, and the length of pauses, which indicated signs of discomfort, anger, or frustration during the data collection process. Data collection from participants proceeded until thematic saturation was achieved with 13 participants, defined as the point at which no new information or emergent themes were observed (Braun, Clarke & Hayfield 2022:432; Kiger & Varpio 2020:2). Each interview lasted for 30 min – 35 min.

### Data analysis

**FIGURE 1:**
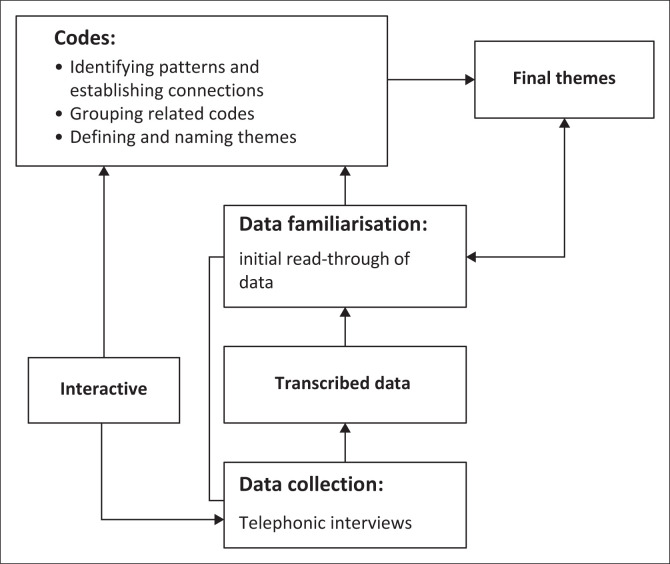
Qualitative data analysis process.

The data from this study were manually analysed using a Microsoft Excel spreadsheet (Redmond, Washington, United States), following the six-step thematic approach (Braun et al. 2022:432) (Figure 1). The data were transcribed verbatim. Transcripts from the interviews were meticulously reviewed by the first author under the guidance of the study supervisors G.H.v.R. and M.M.C. Therefore, the first author N.T. senior researcher familiarised himself with the collected data and re-read all the transcripts. The researcher coded the transcribed data himself as he was more familiar with the study objectives and questions. By utilising the Microsoft document, in vivo codes were generated through a meticulous process of reading each word and line and then highlighting relevant portions of the data using distinct colours. Additionally, the researcher employed concise labels to enhance clarity when referring to specific codes within all the transcripts. Subsequently, the researcher systematically searched, sorted, and compiled all related codes into potential themes based on participants’ thoughts, views, understandings, and interpretations of the nursing care services they received.

Themes were then reviewed for relevance and clarity and were tested for referential adequacy by returning to the raw data. Units of significance were classified and subcategorised based on participants’ point of view, ultimately leading to the identification of themes. The themes were subsequently defined and named, and then synthesised into a cohesive narrative that reflected the participants’ unique stories. An independent coder, holding a Doctorate in Nursing Education and specialising in qualitative research, conducted an autonomous thematic analysis of the data. The findings were then cross-referenced with the primary researcher’s analysis to validate the findings. Participants were uniquely coded from PC1 to PC13, and pseudonyms for telephonic interviews were created followed by a number.

### Trustworthiness

This study employed several strategies to ensure the rigour of its qualitative findings, focusing on credibility, authenticity, confirmability, dependability, and transferability (Amin et al. 2020:1478; Enworo 2023:378; Jamieson, Govaart & Pownall 2023:2; Lincoln & Guba 1985:290). To ensure credibility, interviews were recorded, and the first author N.T. worked under the close guidance of two experienced qualitative researchers G.H.v.R. and M.M.C., who served as research supervisors. As suggested in prior studies, credibility was further ensured through a systematic, repetitive, and recursive process of data analysis and by fostering a trusting relationship with participants during appointments and data collection (Amin et al. 2020:1473; Johnson, Adkins & Chauvin 2020:142).

The study addressed authenticity by accurately portraying participants’ genuine experiences with nursing services, aligning with Guba and Lincoln’s concept, as cited in Amin et al. (2020:1479). This involved selecting participants who had used nursing services, prioritising fairness through informed consent, building strong rapport, asking compassionate and identical questions, and meticulously recording and transcribing data.

Confirmability was achieved through guided decision-making during data analysis using notes and colour-coded texts. Member-checking, researcher confirmation of findings, consultation, detailed documentation, and a relevant literature review all ensured that participants’ views were accurately reflected. Moreover, reflexivity was crucial to ensure confirmability, with researchers documenting their assumptions that could influence data collection. For dependability, consistent themes were identified across all transcripts. An audit trail, outlining all procedures from project initiation through guideline development and validation, was maintained to ensure the study’s reproducibility in different contexts (Lemon & Hayes 2020:608). Transferability was ensured by thoroughly explaining the methodology, allowing other researchers to replicate the study with different populations and in other contexts.

### Ethical considerations

Ethical approval was obtained from the University of South Africa (UNISA). Ethical clearance was granted by the College of Human Sciences Research Ethics Committee (CREC) (Ref no: 51856379_CREC_CHS_2024) and the Executive Director of the Ministry of Health and Social Services (Ref 17/3/3/NPT). Written informed consent was obtained from all participants after they were fully informed about the study’s aims, objectives, benefits, and risks. All participants were made aware of their right to withdraw from the study without facing any consequences. No names or identifying information were required to ensure confidentiality. Data collection respected participants’ privacy by allowing telephonic interviews in the comfort of their homes. This study was conducted in strict adherence to the ethical principles outlined in the Declaration of Helsinki, the internationally recognised ethical guidelines for research involving human subjects.

## Results

### Participant’s demographic data

Table 1 shows the demographic data for the 13 interviewed participants, aged between 25 and 57 years. The sample consisted of eight females and five males, with a mean age of 26. With regard to educational attainment, six of the nine participants with junior or senior secondary certificates were unemployed, compared with two of the four participants with a diploma or degree.

**TABLE 1:** Clients’ demographic profile.

Participant code	Gender	Age (years)	Race	Qualifications	Employment status
PC1	Female	31	Black people	Diploma and/or Degree	Employed
PC2	Female	46	Black people	Junior or Secondary certificate	Unemployed
PC3	Female	35	Black people	Diploma or Degree	Employed
PC4	Male	51	Black people	Junior or Secondary certificate	Employed
PC5	Female	33	Black people	Junior or Secondary certificate	Employed
PC6	Male	40	Black people	Junior or Secondary certificate	Unemployed
PC7	Female	25	Black people	Junior or Secondary certificate	Unemployed
PC8	Female	36	Black people	Junior or Secondary certificate	Unemployed
PC9	Male	28	Black people	Diploma or degree	Unemployed
PC10	Female	50	Black people	Junior or Secondary certificate	Unemployed
PC11	Male	32	Black people	Diploma or Degree	Unemployed
PC12	Female	27	Black people	Junior or Secondary certificate	Employed
PC13	Female	35	Black people	Junior or Secondary certificate	Unemployed

PC, Participant code.

### Themes and subthemes

Two primary themes emerged regarding clients’ perceptions of nursing care services: (1) nurse–client communication, which highlighted disrespectful communication, lack of confidentiality, and lack of enthusiasm for serving clients; and (2) clients’ innovative suggestions to promote nursing care satisfaction, including customer care training and client involvement in decision-making (Table 2).

**TABLE 2:** Themes and subthemes.

Themes	Subthemes
1. Nurse-client communication	1.1. Disrespectful communication 1.2. Lack of client confidentiality 1.3. Lack of enthusiasm to serve
2. Clients’ innovative suggestions to promote nursing care satisfaction	2.1. Customer care training 2.2. Clients’ involvement in decision-making

#### Theme 1: Nurse – client communication

Communication emerged as the primary theme from clients’ responses. This theme focused on clients’ satisfaction with the services provided by selected public health facilities in Namibia. Participants’ accounts suggest that communication is not merely an interpersonal skill, but a critical indicator of relational care, professional identity, and ethical practice. It stemmed from clients’ consistent expressions of disappointment regarding the conduct of nurses, characterised by disrespect, breaches of confidentiality, and apparent disengagement from caring responsibilities. These experiences point to a misalignment between the expected standards of professional nursing conduct and the realities encountered by clients in clinical settings. Within this theme, three key subthemes were identified: disrespectful communication, lack of client confidentiality, and lack of enthusiasm to serve.

**Subtheme 1.1: Disrespectful communication:** The care provided by the nurses did not meet the expectations of the clients, who reported negative behaviour. One issue that emerged from the clients’ responses was that nurses tended to shout. Participants recounted incidents where nurses belittled clients through verbal abuse and shouting, using derogatory terms such as ‘you’, ‘hey’, and ‘shut up’ during communication. From an interpretive perspective, such communication practices can be understood as forms of symbolic power that reinforce hierarchical relationships between nurses and clients. The use of authoritative or dismissive language positions the nurse as dominant and the client as subordinate, thereby undermining the principles of mutual respect and partnership central to contemporary nursing practice. This is particularly concerning in contexts where patients may already feel vulnerable due to illness or socio-economic circumstances:

‘mmmh, [*angry tone*], to say the least it is not what I expect from professional people. Using the word such as “hey you, shup up” and also referring to people as “you” instead of addressing me by my name is an insult.’ (PC7, 25-year-old, female)

‘I am not satisfied with nurses’ shouting and scolding of people in public. I was once addressed by my illness. Mostly the younger nurses are rude toward clients and they are ever on lunch …’ (PC1, 31-year-old, female)

‘Some nurses have persistent misbehaviour goes unpunished. This has potential to create an impression that those who joining nursing will follow those with bad behaviours. Nurses sometimes gossip about their clients’ sickness with unauthorised persons … leaking of this information often come from those closer or related to those nurses.’ (PC13, 35-year-old, female)

**Subtheme 1.2: Lack of client confidentiality:** Confidentiality of clients’ information was a recurring concern raised by participants. Confidentiality is a core ethical principle in nursing practice, essential for maintaining trust and safeguarding clients’ dignity. When this principle is violated, it not only harms individual clients but also erodes confidence in the healthcare system more broadly. Many clients expressed the belief that health complaints and diagnoses shared with a nurse should remain private between the nurse and the client. However, several participants reported instances where nurses disclosed confidential health information to other individuals. The reported disclosure of sensitive information to unauthorised individuals suggests gaps in ethical awareness, professional accountability, or both:

‘I don’t trust some nurses, especially those who are alcoholic [*angry tone*] once drunk they share clients’ illness with their friends.’ (PC5, 33-year-old, female)

‘Nurses sometimes gossip about their clients’ sickness [*illnesses*] with unauthorised persons … leaking of this information often comes from those closer or related to those nurses.’ (PC3, 35-year-old, male)

‘I was really shocked to hear that some nurses reveal client’s health information in public. As we were seated the nurse announced that patients [*clients*] who came for family planning injection must return into two days as there is no stock. Those who came for those particular services appeared embarrassed.’ (PC4, 51-year-old, female)

**Subtheme 1.3: Lack of enthusiasm to serve:** The study’s findings indicate that clients held a negative perception of the enthusiasm shown by nurses. Many participants noted that several nurses seemed uninterested and neglected their needs, while others felt they had to plead or beg for assistance. This perceived disinterest was not expressed as isolated incidents but rather as a recurring pattern across multiple accounts:

‘I believe some nurses pretend to be busy and purposefully ignore to entertain a client request. These are people who lack commitment to serve others.’ (PC2, 46-year-old, female)

In addition to perceived avoidance, participants highlighted the absence of warmth and approachability in nurse–client interactions. Descriptions such as ‘unapproachable’ and the absence of a ‘warm smile’ were used to convey emotional distance, making it difficult for clients to initiate communication or feel at ease in the care environment. For some, this lack of friendliness created a barrier to expressing concerns or seeking clarification:

‘Uuuh, [*pausing*] Many nurses, unfortunately, do not always provide a fulfilling service to their patients. Often, they come across as unapproachable and rarely offer a warm smile to those under their care. This lack of warmth and friendliness can make it challenging for patients to feel comfortable opening up to them.’ (PC1, 31-year-old, female)

Participants also described situations in which obtaining assistance required repeated requests, escalating to emotional distress. The need to ‘beg’, ‘ask over and over’, or even cry before receiving attention was reported as particularly distressing. These accounts reflect experiences where care was perceived as delayed until clients reached a point of visible desperation:

‘It is heartbreaking because you find yourself literally begging for basic help. You have to ask over and over, sometimes even crying, just to get a nurse to listen to you. It feels like you are a burden to them, and they only come to your bed when they see you have run out of options and are pleading for their mercy.’ (PC8, 36-year-old, female)

Ultimately, these findings suggest that the breakdown in communication is not merely a lack of information exchange but is deeply rooted in a perceived erosion of professional conduct and a lack of interpersonal empathy, which significantly diminishes the client’s sense of dignity within the clinical space.

#### Theme 2: Clients’ innovative suggestions to promote client satisfaction

The second theme that emerged from client interviews focused on their recommendations for improving their experiences in public health facilities. Participants not only recounted negative experiences but also articulated specific, and at times detailed, proposals for enhancing nursing care. These suggestions were often framed in direct relation to their prior encounters with nurses, highlighting areas where participants felt improvements were necessary to foster more satisfactory interactions.

Across the interviews, clients demonstrated an awareness of both interpersonal and procedural aspects of care. Their recommendations focused on strengthening relational engagement, improving communication practices, and creating opportunities for more inclusive participation in care processes. Two subthemes that were identified include providing customer care training and involving clients in the decision-making process.

**Subtheme 2.1: Customer care training:** While maintaining emotional control is considered essential for effective customer care, participants expressed concerns about the quality of customer care provided by nurses. Their accounts indicate that client satisfaction was closely associated with how nurses managed interpersonal interactions, particularly in emotionally charged or vulnerable situations. Concerns were raised about nurses’ ability to regulate their emotions, communicate respectfully, and maintain a professional demeanour during client encounters.

Some participants described experiences in which perceived deficiencies in customer care resulted in interactions that felt dismissive or belittling. These experiences were linked to difficulties in establishing open communication, as clients reported feeling discouraged from sharing information or engaging fully in their care:

‘I am not impressed at all. I expect nurses to be the most understanding people but [*a*] lack of customer service [*skills allow*] some nurses to belittle people and inhibits clients from opening up with nurses during their care … I recommend customer care training.’ (PC4, 51-year-old, male)

To address these issues, they recommended that nurses attend courses on customer care to enhance client satisfaction with nursing care services. Some participants suggested that nurses be capacitated to control their emotions, an attribute important in dealing with difficult clients.

‘I think some nurses need anger management courses and or customer care training. Many times nurses lose their temper too easily.’ (PC11, 32-year-old, female)

Participants also referred to a perceived inconsistency between expected professional conduct and observed behaviours. References to the ‘professional oath’ suggest that clients hold clear expectations regarding the ethical and interpersonal standards of nursing practice. Within this context, customer care training was described as a way to reinforce respectful and compassionate engagement:

‘Unfortunately, many nurses exhibit behaviours that conflict with their professional oath. There is a clear need for intensive customer care training to ensure patients are treated with consistent respect and compassion …’ (PC1, 31-year-old, female)

**Subtheme 2.2: Clients’ involvement in decision-making:** Participants also expressed a desire for greater involvement in decisions related to their care. Their accounts suggest that current interactions may not consistently provide opportunities for clients to ask questions, share their perspectives, or seek clarification before decisions are made. This was described as limiting their understanding of care processes and reducing their sense of participation.

Several participants highlighted the importance of being invited into conversations about their care, particularly through open dialogue and the opportunity to ask questions. Encouraging such engagement was viewed as a way to enhance clarity and mutual understanding:

‘Nurses should encourage clients to share their views or even ask questions regarding their care. This will mean clients can get issues clarified before a decision is made regarding their care.’ (PC5, 33-year-old, female)

Participants emphasised the importance of being listened to. Being heard and having one’s views considered were described as key elements of meaningful involvement in care processes:

‘I suggest it is important for nurses to be good listeners and consider clients’ views in their care.’ (PC9, 28-year-old, male)

Across these accounts, participation in decision-making was described in terms of dialogue, listening, and mutual exchange. These suggestions reveal a shift in client expectations, in which individuals no longer view themselves as passive recipients of care but as active stakeholders seeking a collaborative and ‘customer-centric’ healthcare experience that prioritises respect and shared agency.

## Discussion

The study examined clients’ perceptions of nursing care services provided by selected public health facilities in Namibia. During individual interviews, clients expressed frustration with the behaviour of nurses. Participants reported instances of nurses verbally belittling and psychologically abusing clients through infringement on dignity, such as shouting and name-calling. This aligns with the findings of Dzomeku et al. (2020:5), who identified cases of verbal, physical, and psychological abuse directed at service users. There has been an increasing number of reports regarding practices deemed abusive towards clients (Dzomeku et al. 2020:1). Evidence suggests that some nurses may engage in such behaviour for personal gratification, while others have been accused of dehumanising clients (Usberg et al. 2021:1).

Client confidentiality is fundamental to the nursing profession’s values (Ceylan & Çetinkaya 2020:290; Poorchangizi et al. 2019:1). However, participants consistently reported instances where nurses disclosed confidential health information to unauthorised individuals through gossip. Despite the legal obligation of nurses to safeguard clients’ physical, personal, and psychological safety (Cleary & Lees 2019:1) and to protect sensitive medical information (Bani Issa et al. 2020:2), participants raised serious concerns about the conduct of some nurses. They specifically noted that certain nurses engage in gossip and disclose confidential information about their clients. This behaviour not only breaches ethical standards within the nursing profession but also jeopardises the privacy and well-being of clients. Such actions can be rightly classified as unethical behaviour, as argued by Muhsin (2022:1), and may lead to punitive measures if reported to the relevant authorities. Consequently, Poorchangizi et al. (2019:1) have stressed the importance of reinforcing confidentiality, especially given the rising incidence of unethical conduct.

Participants also reported that several nurses appeared uninterested, ignored them and did not provide fulfilling service to their patients. The findings of this study indicated that nurses were not willing to assist or serve their clients with enthusiasm. This lack of enthusiasm may be attributed to emotional exhaustion and stress among nurses (Nobre et al. 2019:1; Parchani et al. 2021:1). Investigating and addressing the motivational challenges faced by nurses is a crucial step towards improving the situation. Future studies should focus on the factors influencing the motivation of nurses in Namibia’s public health sector.

To address the identified challenges, clients suggested focusing on training courses in customer care, areas where nurses need to improve their delivery of care and involve clients in the decision-making process. The emphasis on customer care is increasingly becoming a priority within healthcare systems, aimed at enhancing client outcomes and satisfaction (Fix et al. 2018:301). A key recommendation for the MoHSS is to mandate that all nurses attend training in customer care. The findings of this study are significant, as one of the major challenges faced by healthcare practitioners is understanding clients as active consumers of healthcare services (Leonard & Needham 2020:821). The focus on customer care intends to establish a more client-centred healthcare system, where clients feel valued and supported throughout their healthcare journey (Juanamasta et al. 2019:1). Healthcare providers continually seek ways to enhance customer care by increasing access to services, improving communication with clients, and ensuring that client needs are addressed promptly (Gonzalez 2019:1). By prioritising customer care, healthcare systems can improve client outcomes, reduce costs, and enhance the overall quality of care. While customer care has traditionally been associated with the hospitality industry, there is a growing demand for improved services that respect clients’ rights and raise their expectations (Kurne 2020:13).

These suggestions indicate that customer care training is seen as an effective strategy for enhancing client satisfaction with care (Gonzalez 2019:1). Nguyen, Tran and Nguyen (2021:2525) posited that customer care services significantly improve client care ratings, adherence, health outcomes, and overall quality of care. Furthermore, consistent with the literature, participants in this study indicated that nurses should exhibit strong communication skills, empathy, self-regulation, and patience (Kurne 2020:13). The significance of client involvement in healthcare decision-making cannot be overstated. Clients have expressed a desire to participate in decisions regarding their care. Such involvement is a fundamental aspect of client-centred care and a primary goal for healthcare organisations (Bombard et al. 2018:2). By actively listening to and engaging clients in the decision-making process, healthcare providers can ensure that their clients receive the highest quality of care possible (Rose, Soundy & Rosewilliam 2019:1). Clearly, this study has highlighted that clients want to be actively involved in the decisions related to their care. This trend is indicative of a growing awareness and interest among clients in making informed choices about their healthcare needs. This finding was supported by participants’ reiteration of the need for their right to make decisions to be respected.

### Strengths, limitations, and areas of further research

While the research provides valuable insights into client satisfaction at public health facilities in Namibia, the findings primarily reflect the perceptions of clients within the specific facilities studied. There may have been instances where the researcher’s influence affected the results, making it essential to address potential biases. By acknowledging and reflecting on researchers’ reflexivity, the study’s trustworthiness and credibility were enhanced.

Due to COVID-19 restrictions still in place at the time of data collection, the use of telephonic methods during individual interviews may have affected data quality by limiting the ability to capture field notes, thereby excluding valuable information regarding participants’ body language. Future research should explore client satisfaction through larger cross-sectional or longitudinal studies to formulate strategies aimed at enhancing client satisfaction with nursing care.

### Implications for nursing practice

The study findings have significant implications for nursing practice in Namibia. The consistent reports of disrespectful communication, breaches of confidentiality, and a lack of enthusiasm among nurses highlight a critical need for interventions focused on improving nurse–client interactions. Specifically, the client identified the need for customer care training and professional development opportunities that emphasise communication skills, empathy, and ethical conduct. Furthermore, the expressed desire for client involvement in decision-making underscores the importance of fostering a patient-centred approach to care, in which nurses actively solicit and incorporate clients’ perspectives into treatment plans. These findings call for a multi-pronged approach that includes implementing regular in-service training and promoting a culture of respect, confidentiality, and patient empowerment within healthcare facilities. Investigating and addressing the motivational challenges faced by nurses is a crucial step towards improving the situation. Future studies should focus on the factors influencing the motivation of nurses in the public health sector in Namibia.

## Conclusion

This study revealed significant issues with the quality of nursing care provided, specifically regarding nurses’ disrespectful behaviour, breaches of confidentiality, and their lack of enthusiasm. Collectively, these findings foreground the central role of relational and ethical dimensions of care in shaping clients’ experiences within public health facilities. In response, participants’ suggestions underscore the importance of strengthening interpersonal competencies through contextually relevant customer care training, as well as creating opportunities for more meaningful client involvement in decision-making processes. These proposed areas of improvement reflect clients’ expectations for care that is not only clinically competent but also respectful, responsive, and inclusive. The findings point to a need for sustained, systemic changes to improve the patient experience, professional practice, and the care environment in which care is delivered, and to build a more trusting relationship between healthcare providers and clients.
